# Supplementary material for: Multi-omic analysis reveals lipid dysregulation associated with mitochondrial dysfunction in parkinson’s disease brain
Source: Nat Commun. 2025 Nov 25;16:10490. doi: 10.1038/s41467-025-65489-2 (PMC12647800; doi:10.1038/s41467-025-65489-2)
Supplement: Supplementary file 1 — Supplementary Information [file 41467_2025_65489_MOESM1_ESM.pdf]

Supplementary information associated with the paper *Multi-Omic Analysis Reveals Lipid Dysregulation Associated with Mitochondrial Dysfunction in Parkinson's Disease Brain*

Supplementary Results

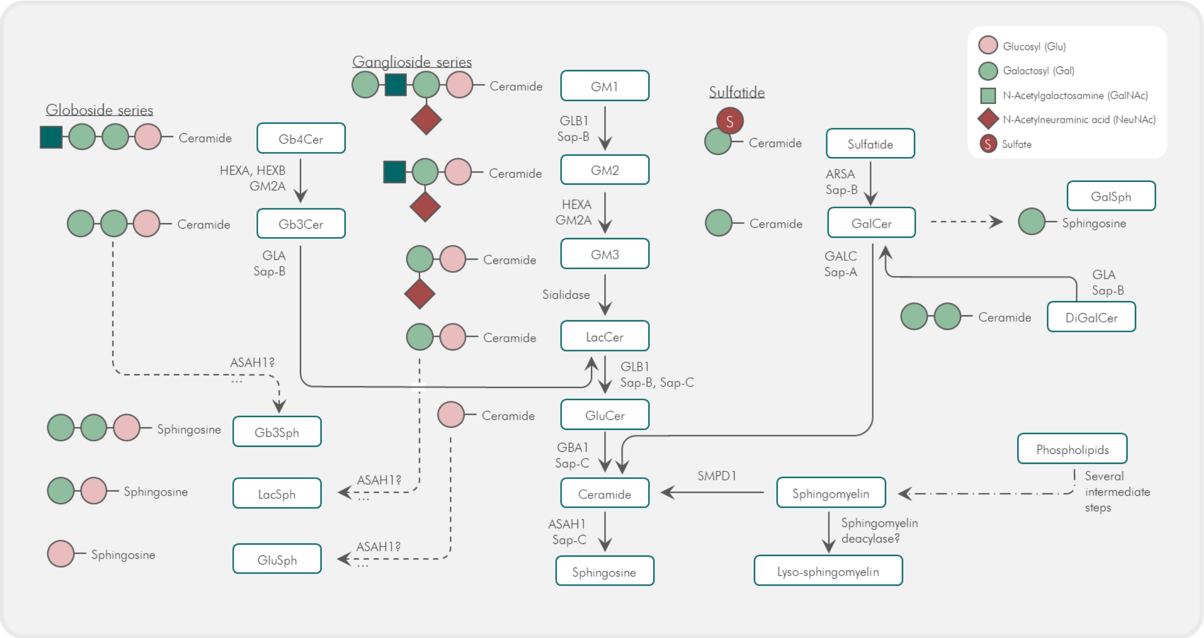

Supplementary Results Figure S1. The catabolic lysosomal glycosphingolipid degradation pathway. *The pathway shows lipids from the globoside, ganglioside and sulfatide series and the enzymes catabolising each step. The diseases associated with malfunctioning of the enzymes are also shown.*

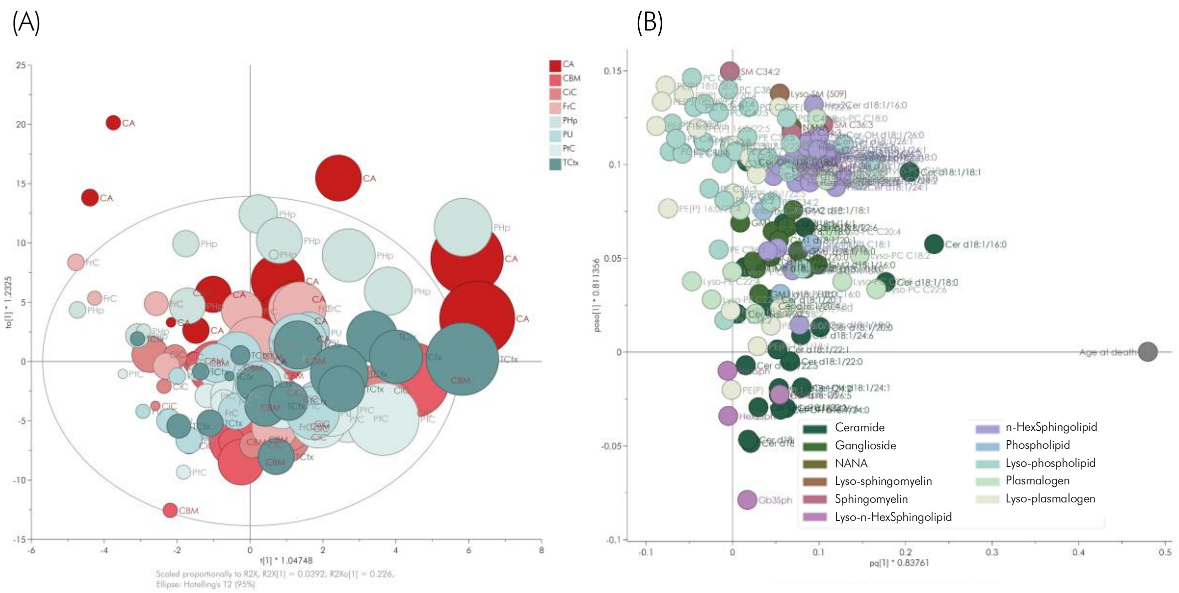

Supplementary Results Figure S2. Relationship between age and lipids in control samples. *(A) OPLS scores from the evaluation of the correlation between age and lipids in controls, sized by age. The OPLS model with age set as the dependent variable was found significant with ANOVA $p = 1.9\ E^{-6}$ and permutations $p << 0.001$ thereby indicating an age effect on the brain lipid levels. (B) OPLS loadings from the evaluation of the correlation between age and lipids in controls. Ceramides with C16 and C18 fatty acid chains showed the strongest correlation with increasing age, along with several n-hexosylceramide species. Source data are provided as a Source Data file.*

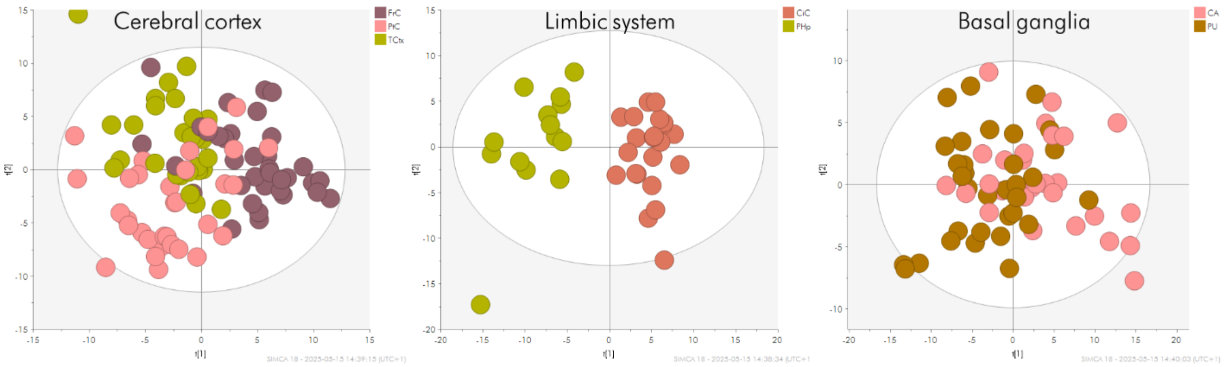

Supplementary Results Figure S3. Regional differences within anatomical brain structures. *Principal components 1 and 2 from principal component analysis of the regional structures cerebral cortex (left), limbic system (middle), and basal ganglia (right). In all regional structures, it was possible to distinguish between the individual regions. Source data are provided as a Source Data file.*

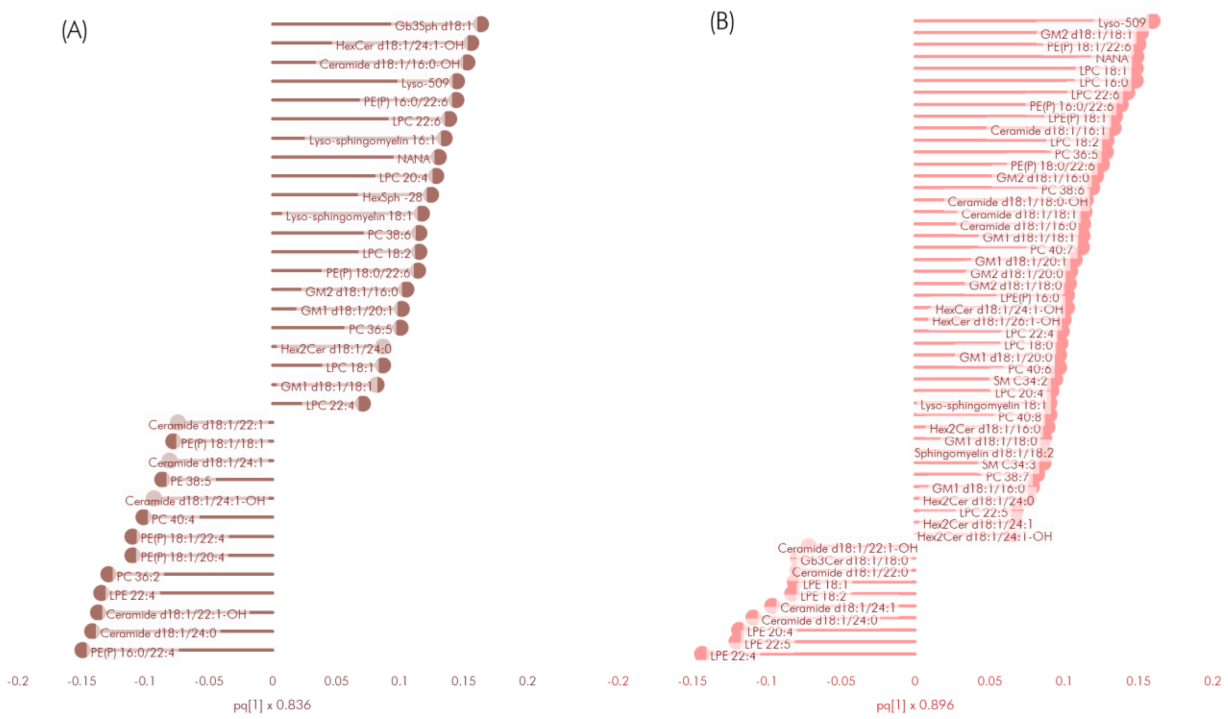

Supplementary Results Figure S4. Significant predictive loadings from OPLS-DA models of mid- and late-stage PD versus control. *(A) Significant loadings from mid-stage PD versus control, and (B) significant loadings from late-stage PD versus control. Negative values represent lipids elevated in controls, while positive values represent lipids elevated in mid- or late-stage PD. Source data are provided as a Source Data file.*

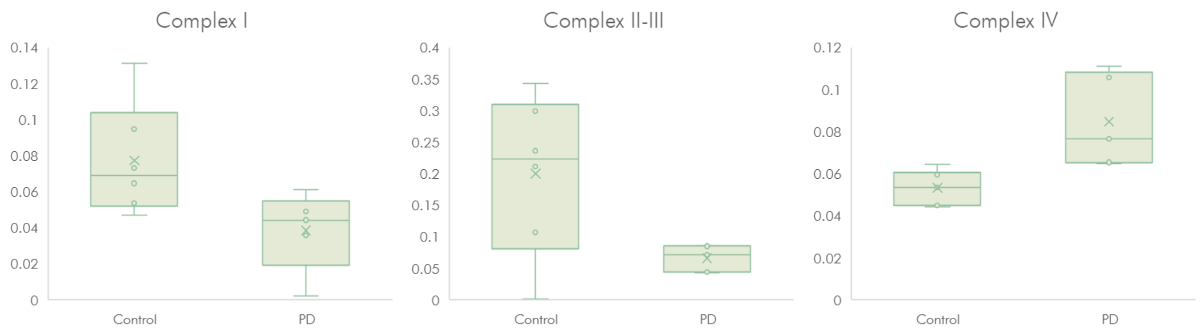

Supplementary Results Figure S5. Ratios of complexes I, II-III and IV to citrate synthase in the putamen region. *All the complex ratios demonstrated nominally significant differences between PD (n = 6) and control (n = 5) when compared by two-sided Student's t-test, with p-values of 0.044, 0.045, and 0.0097 for Complex I, II-III and IV, respectively. Source data are provided as a Source Data file.*

Supplementary Results Table S1. p-values showing if the slope of a linear regression of lipid levels versus age was significantly non-zero. *From the region-wise linear regression of lipids and age at the time of death in all samples. The significantly non-zero slopes (nominal, two-sided Student's t-test) and were age-adjusted. Remaining and non-affected compounds were not age-adjusted.*

	Caudate	Cerebellum	Cingulate cortex	Frontal cortex	Parahippo-campus	Parietal cortex	Putamen	Temporal cortex
Cer d18:1/16:0	4.9E-01	3.4E-02	5.4E-02	7.0E-01	4.8E-03	9.4E-01	5.6E-01	1.4E-01
Cer d18:1/16:1	9.5E-01	9.3E-04	6.7E-02	5.5E-01	1.3E-01	8.8E-01	5.0E-01	1.8E-01
Cer d18:1/18:0	4.8E-01	3.5E-02	1.8E-01	1.6E-01	7.9E-02	6.8E-01	7.3E-01	5.6E-01
Cer d18:1/18:1	4.9E-01	1.9E-03	1.3E-03	1.7E-01	1.9E-02	2.2E-01	8.3E-01	1.5E-03
Cer d18:1/20:0	4.2E-01	8.7E-01	7.5E-01	3.4E-01	5.7E-01	5.0E-01	9.8E-01	7.2E-01
Cer d18:1/20:1		2.3E-02	5.9E-01		3.0E-01			8.9E-01
Cer d18:1/20:4		2.6E-01	8.1E-01		1.7E-01			6.2E-01
Cer d18:1/20:5		7.4E-02	8.2E-01		8.7E-01			3.6E-01
Cer d18:1/22:0	2.7E-01	2.1E-01	5.7E-01	3.1E-01	5.3E-01	3.2E-01	7.6E-02	4.1E-01
Cer d18:1/22:1	7.0E-02	6.0E-02	6.7E-01	1.3E-01	5.8E-01	3.3E-01	1.8E-03	9.0E-01
Cer d18:1/22:5		4.7E-01	8.8E-01		9.7E-01			9.2E-01
Cer d18:1/22:6		1.3E-02	6.6E-02		4.9E-01			9.7E-01
Cer d18:1/24:0	2.5E-01	3.1E-02	6.0E-01	1.9E-01	9.3E-01	3.1E-01	1.2E-03	4.9E-01
Cer d18:1/24:1	3.8E-02	9.9E-02	4.1E-01	1.0E-01	7.4E-01	4.4E-01	2.2E-03	7.2E-01
Cer d18:1/24:2		2.7E-01	6.7E-01		7.5E-01			7.2E-01
Cer d18:1/24:6		1.3E-01	5.5E-01		9.1E-01			2.0E-01
Cer d18:1/26:5		5.3E-01	3.4E-01		2.2E-01			2.4E-01
Cer d18:1/26:6		8.8E-03	4.3E-01		8.4E-01			2.5E-01
Cer-OH d18:1/16:0	6.8E-02	8.9E-02	4.1E-01	5.2E-01	8.6E-01	5.0E-01	4.9E-01	7.3E-01
Cer-OH d18:1/18:0	6.2E-01	1.2E-01	4.1E-01	4.1E-01	2.1E-02	8.5E-01	8.8E-01	8.5E-01
Cer-OH d18:1/22:1		2.3E-01	4.9E-01		8.9E-01			5.7E-01
Cer-OH d18:1/24:0	2.4E-01	3.2E-03	4.7E-01	1.2E-01	5.4E-01	1.8E-01	5.2E-02	5.7E-01
Cer-OH d18:1/24:1	4.5E-01	2.2E-02	3.1E-01	6.3E-02	2.0E-01	6.2E-01	6.3E-02	5.5E-01
GM1 d18:1/16:0	5.1E-01	4.2E-01	1.3E-01	4.3E-02	5.9E-01	5.4E-02	6.3E-01	2.4E-03
GM1 d18:1/18:0	2.5E-01	4.6E-01	1.3E-01	1.2E-02	3.3E-02	1.5E-01	1.9E-01	1.3E-02
GM1 d18:1/18:1	4.5E-01	1.4E-01	1.2E-01	1.7E-03	4.2E-01	3.6E-01	2.3E-01	7.4E-03
GM1 d18:1/20:0		6.5E-01	7.9E-02		5.6E-02			1.4E-02
GM1 d18:1/20:1		2.3E-01	1.2E-01		4.1E-01			9.8E-03
GM1 d18:1/22:0		7.8E-01	1.5E-01		6.5E-01			1.2E-01
GM1 d18:1/24:1	5.2E-01	2.3E-01	2.7E-01	8.7E-01	2.2E-02	1.5E-02	2.0E-01	2.5E-02
GM2 d18:1/16:0	3.9E-02	3.6E-01	1.9E-01	1.6E-03	6.7E-02	4.3E-01	5.2E-01	4.2E-04
GM2 d18:1/18:0	3.2E-01	3.7E-01	1.5E-01	2.3E-02	9.1E-01	1.6E-01	5.9E-01	3.8E-02
GM2 d18:1/18:1	3.8E-01	1.1E-01	7.1E-02	2.4E-02	3.9E-01	3.2E-01	9.4E-01	1.1E-02
GM2 d18:1/20:0		6.3E-01	8.5E-02		8.6E-01			5.9E-02
GM3 d18:1/16:0	6.3E-01			5.1E-01		6.5E-01	3.3E-01	
GM3 d18:1/18:0	8.3E-01	9.7E-01	3.3E-02	1.4E-02	6.2E-01	3.2E-01	3.7E-01	2.7E-02
GM3 d18:1/18:1	5.8E-01			6.2E-01		5.0E-01	8.9E-01	
GM3 d18:1/24:1	9.5E-01			6.3E-01		9.2E-01	8.1E-01	
Gb3Sph d18:1	5.0E-02	4.6E-01	5.0E-01	3.3E-01	7.5E-01	3.3E-01	3.5E-01	1.3E-01
Hex2Sph d18:1	6.5E-01	2.0E-01	5.1E-01	4.2E-02	2.7E-01	3.0E-01	6.1E-03	3.1E-01
HexSph -28	7.9E-02	7.1E-01	1.1E-01	1.6E-01	5.6E-01	9.1E-01	1.1E-01	3.3E-02

HexSph d18:1	8.1E-01	8.2E-02	9.6E-01	1.1E-01	9.5E-01	8.8E-01	1.3E-01	4.7E-01
Lyso-PC C16:0	9.4E-01	2.9E-01	5.0E-02	6.6E-01	4.8E-02	2.0E-01	4.7E-01	1.2E-01
Lyso-PC C18:0	9.9E-01	5.2E-01	1.0E-01	9.3E-01	2.2E-01	9.4E-02	4.9E-02	5.3E-02
Lyso-PC C18:1	9.3E-01	1.1E-01	4.0E-02	6.1E-01	1.9E-01	1.3E-01	6.0E-01	4.8E-01
Lyso-PC C18:2	9.4E-01	7.1E-01	1.4E-01	9.7E-01	3.0E-01	5.1E-01	2.9E-01	2.6E-01
Lyso-PC C20:4	6.2E-01	4.5E-01	6.6E-02	4.5E-01	2.5E-01	8.1E-01	3.7E-01	2.6E-01
Lyso-PC C22:4	9.7E-01	2.6E-01	1.3E-01	1.0E+00	9.2E-01	2.9E-01	4.2E-01	5.7E-01
Lyso-PC C22:5		1.8E-01	4.9E-02		2.1E-01			1.4E-01
Lyso-PC C22:6	7.2E-01	2.2E-01	5.8E-03	8.9E-01	2.8E-01	3.3E-01	1.6E-01	2.1E-01
Lyso-PE C16:0	6.7E-01	1.1E-01	4.0E-01	6.8E-01	8.2E-01	5.3E-02	1.1E-01	5.0E-01
Lyso-PE C18:0	6.5E-01			7.6E-01		1.9E-01	1.0E-02	
Lyso-PE C18:1	7.5E-01	6.6E-02	7.0E-01	1.6E-01	8.3E-01	5.4E-01	3.8E-03	4.9E-01
Lyso-PE C18:2	9.3E-02	1.2E-01	4.6E-01	6.8E-01	5.1E-01	5.7E-01	1.7E-01	9.6E-02
Lyso-PE C20:4	2.3E-01	3.1E-01	9.5E-01	8.6E-01	7.8E-01	2.3E-01	3.9E-01	8.7E-01
Lyso-PE C20:5	4.5E-01			4.0E-01		7.3E-01	2.5E-01	
Lyso-PE C22:4	1.3E-01	3.0E-01	1.7E-01	1.6E-01	4.4E-02	6.6E-01	1.4E-02	3.9E-01
Lyso-PE C22:5	3.0E-01	3.7E-01	5.9E-01	9.3E-01	5.6E-01	9.6E-01	4.8E-01	1.7E-01
Lyso-PE C22:6	1.7E-01	4.0E-01	8.6E-02	4.9E-01	5.8E-01	6.6E-01	5.5E-01	3.9E-01
Lyso-PE C(P) 16:0	4.4E-01	3.9E-01	9.9E-01	1.9E-01	2.7E-01	9.7E-01	7.7E-01	2.0E-01
Lyso-PE C(P) 16:1	5.5E-01			9.4E-01		4.7E-01	8.4E-01	
Lyso-PE C(P) 18:0	3.8E-01	5.2E-01	3.1E-01	8.3E-01	7.5E-02	3.7E-01	2.6E-02	4.8E-02
Lyso-PE C(P) 18:1	8.5E-01	7.2E-02	8.3E-01	3.5E-01	5.1E-01	8.4E-01	1.1E-05	3.0E-01
Lyso-509	6.9E-01	1.9E-01	6.4E-01	4.2E-01	9.4E-02	2.3E-01	5.9E-01	1.1E-01
Lyso-SM 16:1		3.5E-02	6.2E-04		8.1E-02			2.2E-01
Lyso-SM 18:1	9.0E-01	4.1E-01	1.0E-01	5.6E-01	9.9E-02	1.9E-01	8.2E-01	1.9E-01
NANA		2.0E-01	3.9E-01		3.9E-01			2.5E-01
Gb3Cer d18:1/16:0		8.1E-01	1.1E-01		2.5E-01			6.1E-02
Gb3Cer d18:1/18:0		5.4E-02	3.2E-01		9.5E-02			7.5E-01
Gb4Cer d18:1/16:0	4.0E-02	5.5E-01	2.6E-02	4.0E-02	3.8E-01	3.2E-01	7.8E-01	4.0E-02
Hex2Cer d18:1/16:0	2.5E-01	5.8E-01	5.3E-01	8.6E-01	9.4E-03	4.7E-01	2.0E-02	1.3E-01
Hex2Cer d18:1/18:0	8.2E-01	8.3E-01	1.5E-01	6.5E-01	1.3E-03	5.5E-01	9.6E-02	4.4E-02
Hex2Cer d18:1/24:0	2.5E-02	8.5E-01	1.2E-01	5.0E-01	2.7E-03	6.1E-01	4.6E-01	3.0E-03
Hex2Cer d18:1/24:1	4.1E-02	7.7E-01	1.8E-01	4.9E-01	3.9E-03	3.9E-01	3.4E-01	4.8E-03
Hex2Cer d18:1/24:1-OH		4.1E-01	1.1E-01		2.6E-03			7.2E-04
Hex2Cer d18:1/24:2-OH		5.4E-01	5.8E-02		4.5E-03			4.1E-03
HexCer d18:1/16:0	6.0E-01	2.2E-01	5.4E-01	2.2E-01	2.2E-01	5.7E-01	3.7E-03	8.0E-01
HexCer d18:1/16:1	8.6E-01	1.5E-01	6.1E-01	1.5E-01	1.7E-01	7.8E-01	7.5E-04	1.8E-01
HexCer d18:1/18:0		6.0E-01	8.0E-01		4.8E-01			9.5E-01
HexCer d18:1/18:0-OH		6.0E-01	4.6E-01		6.3E-02			9.9E-03
HexCer d18:1/18:1	4.6E-01	1.6E-01	3.6E-01	3.8E-01	5.0E-01	8.2E-01	1.6E-04	6.5E-01
HexCer d18:1/20:0-OH		6.7E-01	7.8E-01		5.3E-02			9.5E-03
HexCer d18:1/22:0-OH		6.0E-01	2.8E-01		2.4E-01			1.1E-01
HexCer d18:1/24:0		8.6E-01	6.4E-01		1.9E-01			4.8E-02
HexCer d18:1/24:0-OH	6.2E-02	8.5E-01	1.9E-01	8.2E-02	1.8E-01	4.5E-01	8.9E-01	4.0E-03
HexCer d18:1/24:1		4.1E-01	4.5E-01		1.9E-01			1.4E-01
HexCer d18:1/24:1-OH	3.7E-02	3.1E-01	2.8E-01	7.0E-01	1.2E-01	9.9E-01	2.9E-01	2.4E-03
HexCer d18:1/24:2-OH		4.0E-01	3.4E-01		8.6E-02			1.6E-02
HexCer d18:1/26:0		2.1E-01	1.7E-01		5.7E-02			2.2E-03
HexCer d18:1/26:0-OH		5.5E-01	1.4E-01		1.2E-01			1.2E-03

HexCer d18:1/26:1		6.0E-01	5.0E-01		5.7E-02			1.4E-02
HexCer d18:1/26:1-OH		2.1E-01	1.7E-01		1.0E-01			4.3E-03
PC C34:1	3.6E-01			5.7E-01		4.8E-01	4.7E-01	
PC C34:2	2.9E-01	6.0E-01	7.5E-01	5.5E-01	8.8E-01	6.5E-01	1.2E-01	3.1E-01
PC C36:1	3.4E-01	9.7E-01	2.8E-01	5.2E-01	8.1E-01	5.1E-01	5.2E-01	2.0E-01
PC C36:2	2.4E-01	8.9E-01	3.1E-01	7.1E-01	8.2E-01	5.1E-01	4.6E-02	9.2E-01
PC C36:3	3.6E-01			2.3E-01		2.4E-01	2.7E-01	
PC C36:4	4.2E-01	6.5E-01	9.1E-01	2.6E-01	2.9E-01	4.8E-01	5.3E-01	2.2E-01
PC C36:5	9.1E-01	3.2E-01	1.1E-01	1.2E-01	6.4E-02	2.8E-01	3.2E-01	1.0E-01
PC C38:4	4.4E-01	2.2E-01	7.3E-01	1.4E-01	2.9E-01	4.0E-01	1.0E+00	1.9E-01
PC C38:5	4.5E-01	7.3E-01	9.0E-01	1.9E-01	6.0E-01	2.8E-01	4.6E-01	2.2E-01
PC C38:6	4.3E-01	6.7E-01	1.4E-01	3.2E-01	8.8E-01	2.5E-01	8.9E-01	2.6E-01
PC C38:7	8.6E-01	2.8E-01	4.7E-02	2.1E-01	4.9E-02	1.7E-01	9.4E-01	1.4E-02
PC C40:4	5.1E-01	7.4E-01	3.0E-01	4.5E-02	1.5E-01	2.8E-01	6.7E-01	3.6E-01
PC C40:5	4.0E-01	4.8E-01	7.6E-01	1.1E-01	2.2E-01	2.6E-01	9.9E-01	3.3E-01
PC C40:6	4.3E-01	8.5E-01	2.8E-01	1.3E-01	9.9E-01	1.3E-01	1.8E-01	2.6E-01
PC C40:7	4.7E-01	8.8E-01	2.6E-01	4.2E-01	4.2E-01	1.2E-01	7.0E-01	2.9E-01
PC C40:8	9.0E-01	9.9E-01	9.1E-01	2.3E-01	3.8E-01	2.4E-01	7.1E-01	3.1E-01
PE C34:1	4.2E-01	4.4E-01	6.3E-01	3.0E-01	5.2E-02	1.8E-01	9.1E-01	8.0E-01
PE C34:2	2.9E-01	1.3E-01	1.5E-01	4.3E-01	3.4E-02	5.0E-01	5.2E-04	6.3E-01
PE C36:1	6.4E-01	6.4E-01	8.3E-01	1.2E-01	2.7E-01	9.3E-02	8.6E-02	4.2E-01
PE C36:2	3.3E-01	1.9E-01	6.3E-01	3.1E-01	5.7E-01	9.6E-02	3.6E-03	4.4E-01
PE C36:3	2.0E-01	7.5E-01	7.6E-02	3.0E-01	3.8E-01	1.8E-01	6.0E-02	7.0E-01
PE C36:4	3.6E-01	3.9E-01	5.9E-01	3.3E-02	8.3E-01	3.7E-01	6.2E-01	7.2E-01
PE C36:5	5.0E-01	6.2E-01	1.3E-01	5.2E-02	5.2E-02	2.9E-01	1.4E-01	5.7E-01
PE C38:4	9.1E-01	8.1E-01	3.0E-01	1.5E-01	9.7E-01	5.7E-01	4.9E-01	5.8E-01
PE C38:5	7.9E-01	7.3E-01	1.9E-01	3.4E-01	7.4E-01	4.4E-01	4.4E-02	6.4E-01
PE C38:6	8.6E-01	6.0E-01	3.5E-02	5.2E-01	9.7E-01	3.0E-01	7.5E-01	6.0E-01
PE C38:7	8.6E-01	5.4E-01	3.5E-02	4.8E-01	1.9E-01	4.4E-01	7.6E-01	2.7E-01
PE C40:4	8.7E-01	5.6E-01	3.9E-01	2.5E-01	6.2E-01	3.6E-01	3.1E-01	8.1E-01
PE C40:5	6.1E-01	7.9E-01	9.6E-02	4.6E-01	8.7E-01	3.4E-01	5.5E-01	7.2E-01
PE C40:6	7.3E-01	8.8E-01	5.5E-03	5.0E-01	4.6E-01	1.7E-01	1.9E-01	4.5E-01
PE C40:7	8.5E-01	6.6E-01	1.5E-02	9.1E-01	3.7E-01	2.0E-01	6.3E-01	3.3E-01
PE C40:8	8.1E-01	9.4E-01	1.9E-01	3.7E-01	5.6E-01	3.5E-01	9.5E-01	8.8E-01
PE(P) 16:0/18:1	6.3E-01	9.7E-01	2.3E-01	1.2E-01	7.1E-01	9.2E-01	1.3E-04	6.5E-01
PE(P) 16:0/20:4	9.0E-01	2.8E-01	2.3E-01	2.2E-01	9.6E-01	7.0E-01	3.5E-03	8.8E-01
PE(P) 16:0/22:4	4.9E-01	6.3E-01	1.8E-01	6.2E-01	5.3E-01	8.6E-01	1.2E-03	6.8E-01
PE(P) 16:0/22:5	1.9E-02	2.8E-01	2.2E-01	5.1E-01	8.2E-01	3.3E-01	4.7E-02	3.0E-01
PE(P) 16:0/22:6	5.5E-01	7.3E-01	4.4E-01	8.1E-02	8.8E-01	1.7E-02	7.8E-01	1.6E-01
PE(P) 16:1/18:1	4.4E-01			7.1E-01		1.9E-01	1.1E-01	
PE(P) 16:1/22:4	2.1E-02			3.9E-01		3.1E-01	3.0E-01	
PE(P) 16:1/22:6	1.7E-01			6.7E-01		6.7E-01	4.0E-01	
PE(P) 18:0/18:1	1.2E-01	7.3E-01	1.7E-01	5.2E-01	6.2E-01	2.0E-01	1.5E-01	8.6E-01
PE(P) 18:0/20:4	9.1E-01	8.6E-01	7.4E-01	5.5E-02	6.7E-01	3.0E-01	4.8E-01	6.3E-01
PE(P) 18:0/22:4	7.7E-01	8.7E-01	8.6E-01	4.2E-01	8.2E-01	6.0E-01	9.9E-01	3.8E-01
PE(P) 18:0/22:5	8.6E-01	8.9E-01	5.9E-01	3.0E-01	9.6E-01	3.3E-01	5.6E-01	3.5E-01
PE(P) 18:0/22:6	8.8E-01	7.6E-01	6.8E-02	1.5E-01	4.5E-01	9.5E-02	1.9E-01	1.9E-01
PE(P) 18:1/16:0		2.6E-01	3.1E-01		4.8E-01			8.8E-01
PE(P) 18:1/18:1	3.0E-01	2.1E-01	5.9E-01	1.6E-01	8.0E-01	5.6E-01	1.3E-05	7.4E-01

PE(P) 18:1/20:4	9.1E-01	5.8E-01	2.0E-01	9.8E-01	7.7E-01	6.2E-01	1.5E-05	5.2E-01
PE(P) 18:1/22:4	3.2E-01	1.7E-01	3.5E-01	8.0E-01	9.2E-01	8.9E-01	2.8E-05	7.3E-01
PE(P) 18:1/22:5	7.8E-01	6.7E-02	8.3E-01	7.6E-01	3.9E-01	2.6E-01	2.3E-03	8.4E-01
PE(P) 18:1/22:6	7.0E-01	4.9E-02	2.1E-02	2.1E-01	2.8E-01	1.7E-01	5.1E-01	1.4E-01
SM C34:2		5.1E-01	6.0E-01		6.8E-01			7.0E-02
SM C34:3		2.0E-01	9.9E-03		4.9E-02			1.3E-03
SM C36:3	9.7E-01	8.1E-02	1.2E-02	4.9E-01	4.4E-02	4.1E-01	3.5E-02	3.1E-04

Supplementary Results Table S2. Linear mixed effects model of lipids and the class (PD or control) and mitochondrial activity (complex I, II-III and IV ratioed to citrate synthase) interaction. *The significant interactions post Benjamini-Hochberg multiple testing correction at FDR = 10% are reported. The lipids are represented by the 95% confidence interval, standard error and the non-adjusted p-value. No significant interactions were observed in complex IV.*

	**Complex I/CS**	**95% CI +/- SE**	**Raw p-value**
Complex I/citrate synthase ratio	Cer d18:1/20:1	[-23.6, -5.2 +/- 4.7]	4.7E-03
	Cer d18:1/22:1	[-32.8, -8.4 +/- 6.2]	2.7E-03
	Cer OH d18:1/24:0	[-23.9, -3.4 +/- 5.2]	1.3E-02
	HexCer d18:1/16:0	[-20.9, -4.3 +/- 4.2]	5.8E-03
	HexCer d18:1/18:1	[-23.9, -5.3 +/- 4.7]	4.5E-03
	HexSph	[-42.8, -13.3 +/- 7.5]	8.6E-04
	Lyso-PC C16:0	[5.6, 21.3 +/- 4.0]	2.1E-03
	Lyso-PC C18:2	[7.6, 31.0 +/- 6.0]	3.5E-03
	Lyso-PC C20:4	[5.4, 25.5 +/- 5.1]	7.6E-03
	Lyso-PC C22:6	[6.4, 27.1 +/- 5.3]	1.1E-02
	Lyso-PE(P) C18:1	[-35.3, -10.6 +/- 6.3]	1.1E-03
	PE(P) 16:0/18:1	[-39.2, -13.4 +/- 6.6]	5.5E-04
	PE(P) 16:0/22:4	[-35.3, -5.5 +/- 7.6]	1.3E-02
	PE(P) 18:0/18:1	[-35.3, -9.6 +/- 6.6]	2.2E-03
	PE(P) 18:1/18:1	[-39.8, -11.8 +/- 7.1]	1.2E-03
	PE(P) 18:1/20:4	[-40.7, -10.9 +/- 7.6]	2.1E-03
	PE(P) 18:1/22:4	[-34.8, -7.3 +/- 7.0]	5.6E-03
	PE(P) 18:1/22:5	[-32.3, -7.4 +/- 6.3]	3.8E-03
Complex II-III/citrate synthase ratio			
	Cer d18:1/22:6	[-14.2, -4.6 +/- 2.4]	4.7E-04

Supplementary Methods

Supplementary Methods Table S1. Demographics of samples from controls and Parkinson's disease in eight different brain regions. *Numbers of each group, mean age and standard deviation, percentage of females, and the results from the Benjamini-Hochberg adjusted (FDR = 5%) Student's two-tailed t-test comparing the ages between the groups are presented. The PD samples further consisted of individuals with Braak stage classified as 3, 4, 5 or 6 – divided into "Braak 3 - 4" and "Braak 5 - 6" in the table. In the cases where the number of age observations are only one, the actual age is given in parentheses.*

Region	Class	n	Average age +/- standard deviation	% Females	p-value: Age: Total PD vs control	p-value: Age: Braak 3-4 vs control
Caudate	Control	13	74.3 ± 8.7	54%	NS	NS
	Total PD	15	76.7 ± 8.4	47%		
	Braak 3 - 4	13	76.1 ± 8.8	38%		
	Braak 5 - 6	2	81 ± 1.4	100%		
Cerebellum	Control	14	72 ± 7.8	36%	*	NS
	Total PD	16	78.6 ± 5.9	44%		
	Braak 3 - 4	10	78.6 ± 7	60%		
	Braak 5 - 6	6	78.5 ± 4	17%		
Cingulate cortex	Control	13	72.6 ± 7.7	46%	NS	NS
	Total PD	11	78.9 ± 6.7	64%		
	Braak 3 - 4	10	78.6 ± 7	60%		
	Braak 5 - 6	1	(82.0)	100%		
Frontal cortex	Control	12	70.7 ± 6.8	25%	*	NS
	Total PD	23	78.2 ± 6	35%		
	Braak 3 - 4	16	77.9 ± 6.9	38%		
	Braak 5 - 6	7	78.7 ± 3.7	29%		
Parahippocampus	Control	11	71 ± 6.8	36%	NS	NS
	Total PD	5	79.6 ± 7.7	60%		
	Braak 3 - 4	4	79 ± 8.8	50%		
	Braak 5 - 6	1	(82.0)	100%		
Parietal cortex	Control	15	72.4 ± 7.7	33%	*	NS
	Total PD	17	78.6 ± 6.6	47%		
	Braak 3 - 4	15	78.3 ± 7	40%		
	Braak 5 - 6	2	81 ± 1.4	100%		
Putamen	Control	13	71.6 ± 6.4	38%	NS	NS
	Total PD	20	76.6 ± 8.8	45%		
	Braak 3 - 4	13	75.5 ± 10.6	54%		
	Braak 5 - 6	7	78.7 ± 3.7	29%		
Temporal cortex	Control	16	72.3 ± 7.3	38%	*	NS
	Total PD	11	78.9 ± 6.7	64%		
	Braak 3 - 4	10	78.6 ± 7	60%		
	Braak 5 - 6	1	(82.0)	100%		

Supplementary Methods Table S2. Chromatographic separation parameters for the three analytical LC methods. *The table shows the analytes, column chemistry and temperature, composition of mobile phase A and B, and the timings, flow rate and B percentage of the LC gradient.*

Analytes	Column chemistry	Mobile phase	LC method		
GM1, GM2, GM3, NANA, ceramide	BEH Amide, 1 x 150 mm, 1.7µm<br>Column temperature: 60℃	A: 10 mM NH_4COOH in 85% acetonitrile<br>B: 10 mM NH_4COOH in 15% acetonitrile	Time [min]	Rate[mL/min]	%B
			0	0.2	0
			0.5	0.2	0
			8	0.2	30
			10	0.2	70
			10.5	0.2	80
			11.5	0.2	80
			11.6	0.2	0
			13.3	0.2	0
			15.83	0.2	0
			16	0.2	0
HexCer, Hex2Cer, Gb3Cer, Gb4Cer, HexSph, Hex2Sph, Gb3Sph, SM, lyso-SM	BEH C8, 2.1 x 50 mm, 1.7 µm<br>Column temperature: 50℃	A: 0.1% formic acid in water<br>B: 0.1% formic acid in methanol	Time [min]	Rate[mL/min]	%B
			0	0.5	50
			0.2	0.5	50
			2	0.5	100
			3	0.5	100
			3.1	0.5	50
			5	0.5	50
PE, PC, PE(P), lyso-PE, lyso-PC, lyso-PE(P)	BEH HILIC, 2.1 x 50 mm, 1.7 µm<br>Column temperature: ambient	A: 10 mM NH_4CH_3COOH in 95% acetonitrile,<br>B: 10 mM NH_4CH_3COOH in 50% acetonitrile	Time [min]	Rate[mL/min]	%B
			0	0.5	0
			0.5	0.5	0
			3.75	0.5	20
			3.76	0.5	100
			5.49	0.5	100
			5.5	0.8	100
			5.75	0.8	100
			5.76	0.8	0
			7.5	0.8	0
			7.51	0.5	0
			7.75	0.5	0

Supplementary Methods Table S3. Multiple reaction monitoring transitions and collision energies. *The table shows the transition from precursor to product ion, the cone and collision energies applied, and finally if detection was performed in positive (ESI+) or negative (ESI-) electrospray ionisation mode.*

Compound	Transition	Cone	Collision	Detection mode
Cer d18:1/16:0	536.50 > 280.26	100	30	ESI-
Cer d18:1/16:1	534.49 > 278.25	100	30	ESI-
Cer d18:1/18:0	564.54 > 308.30	100	30	ESI-
Cer d18:1/18:1	562.52 > 306.28	100	30	ESI-
Cer d18:1/20:0	592.57 > 336.33	100	30	ESI-
Cer d18:1/20:1	590.55 > 334.31	100	30	ESI-
Cer d18:1/20:4	584.50 > 328.26	100	30	ESI-
Cer d18:1/20:5	582.49 > 326.25	100	30	ESI-
Cer d18:1/22:0	620.60 > 364.36	100	30	ESI-
Cer d18:1/22:1	618.58 > 362.34	100	30	ESI-
Cer d18:1/22:5	610.52 > 354.28	100	30	ESI-
Cer d18:1/22:6	608.50 > 352.26	100	30	ESI-
Cer d18:1/24:0	648.63 > 392.39	100	30	ESI-
Cer d18:1/24:1	646.61 > 390.37	100	30	ESI-
Cer d18:1/24:2	644.60 > 388.36	100	30	ESI-
Cer d18:1/24:6	636.54 > 380.30	100	30	ESI-
Cer d18:1/26:5	666.58 > 410.34	100	30	ESI-
Cer d18:1/26:6	664.57 > 408.33	100	30	ESI-
Cer-OH d18:1/16:0	552.50 > 296.26	100	30	ESI-
Cer-OH d18:1/18:0	580.53 > 324.29	100	30	ESI-
Cer-OH d18:1/22:1	634.58 > 378.34	100	30	ESI-
Cer-OH d18:1/24:0	664.62 > 408.38	100	30	ESI-
Cer-OH d18:1/24:1	662.61 > 406.37	100	30	ESI-
GM1 d18:1/16:0	1516.84 > 290.09	70	58	ESI-
GM1 d18:1/18:0	1544.87 > 290.09	70	58	ESI-
GM1 d18:1/18:1	1542.85 > 290.09	70	58	ESI-
GM1 d18:1/20:0	1572.90 > 290.09	70	58	ESI-
GM1 d18:1/20:1	1570.88 > 290.09	70	58	ESI-
GM1 d18:1/22:0	1600.93 > 290.09	70	58	ESI-
GM1 d18:1/24:1	1626.95 > 290.09	70	58	ESI-
GM2 d18:1/16:0	1354.78 > 290.09	70	58	ESI-
GM2 d18:1/18:0	1382.82 > 290.09	70	58	ESI-
GM2 d18:1/18:1	1380.80 > 290.09	70	58	ESI-
GM2 d18:1/20:0	1410.85 > 290.09	70	58	ESI-
GM3 d18:1/18:0	1179.74 > 290.09	70	58	ESI-
GM3 d18:1/16:0	1151.71 > 290.09	70	58	ESI-
GM3 d18:1/18:1	1177.72 > 290.09	70	58	ESI-
GM3 d18:1/24:1	1261.81 > 290.09	70	58	ESI-
Gb3Sph	786.48 > 282.36	72	32	ESI+
Hex2Sph	624.40 > 282.36	35	16	ESI+
HexSph -28	434.37 > 236.32	92	16	ESI+

HexSph	462.46 > 264.30	92	16	ESI+
Lyso-PC C16:0	496.34 > 184.07	30	20	ESI+
Lyso-PC C18:2	520.34 > 184.07	30	20	ESI+
Lyso-PC C18:1	522.36 > 184.07	30	20	ESI+
Lyso-PC C18:0	524.37 > 184.07	30	20	ESI+
Lyso-PC C20:4	544.34 > 184.07	30	20	ESI+
Lyso-PC C22:6	568.34 > 184.07	30	20	ESI+
Lyso-PC C22:5	570.36 > 184.07	30	20	ESI+
Lyso-PC C22:4	572.37 > 184.07	30	20	ESI+
Lyso-PE C16:0	454.29 > 313.27	30	20	ESI+
Lyso-PE C18:2	478.29 > 337.27	30	20	ESI+
Lyso-PE C18:1	480.31 > 339.29	30	20	ESI+
Lyso-PE C20:4	502.29 > 361.27	30	20	ESI+
Lyso-PE C22:6	526.29 > 385.27	30	20	ESI+
Lyso-PE C22:5	528.31 > 387.29	30	20	ESI+
Lyso-PE C22:4	530.33 > 389.31	30	20	ESI+
Lyso-PE C18:0	482.32 > 341.31	30	20	ESI+
Lyso-PE C20:5	500.28 > 359.26	30	20	ESI+
Lyso-PE(P) C16:1	436.28 > 264.42	52	24	ESI+
Lyso-PE(P) C16:0	438.32 > 266.46	52	24	ESI+
Lyso-PE(P) C18:1	464.34 > 292.47	42	24	ESI+
Lyso-PE(P) C18:0	466.35 > 294.49	40	24	ESI+
Lyso-SM C16:1	437.31 > 184.07	30	20	ESI+
Lyso-SM C18:1	465.35 > 184.07	30	20	ESI+
PPPCS (Lyso-SM 509)	509.31 > 184.07	30	20	ESI+
NANA	307.95 > 86.81	60	12	ESI-
Gb3Cer d18:1/16:0	1046.63 > 884.66	124	64	ESI+
Gb3Cer d18:1/18:0	1074.68 > 912.71	124	64	ESI+
Gb4Cer d18:1/16:0	1249.74 > 884.68	125	52	ESI+
Hex2Cer d18:1/16:0	884.61 > 722.56	124	57	ESI+
Hex2Cer d18:1/18:0	912.64 > 750.59	124	57	ESI+
Hex2Cer d18:1/24:1	994.72 > 832.66	124	57	ESI+
Hex2Cer d18:1/24:0	996.73 > 834.68	124	57	ESI+
Hex2Cer-OH d18:1/24:2	1008.70 > 846.64	124	57	ESI+
Hex2Cer-OH d18:1/24:1	1010.71 > 848.66	124	57	ESI+
HexCer d18:1/16:1	720.70 > 558.70	125	52	ESI+
HexCer d18:1/16:0	722.67 > 560.64	125	52	ESI+
HexCer d18:1/18:1	748.60 > 586.60	125	52	ESI+
HexCer d18:1/18:0	750.60 > 588.60	125	52	ESI+
HexCer-OH d18:1/18:0	766.60 > 264.30	140	44	ESI+
HexCer-OH d18:1/20:0	794.60 > 264.30	140	44	ESI+
HexCer-OH d18:1/22:0	820.70 > 658.80	140	44	ESI+
HexCer d18:1/24:1	832.70 > 670.70	125	52	ESI+
HexCer d18:1/24:0	834.70 > 672.70	125	52	ESI+
HexCer-OH d18:1/24:2	846.70 > 684.80	140	44	ESI+
HexCer-OH d18:1/24:1	848.70 > 686.80	140	44	ESI+

HexCer-OH d18:1/24:0	850.70 > 688.80	140	44	ESI+
HexCer d18:1/26:1	860.73 > 698.76	125	52	ESI+
HexCer d18:1/26:0	862.70 > 700.80	125	52	ESI+
HexCer-OH d18:1/26:1	876.72 > 714.75	140	44	ESI+
HexCer-OH d18:1/26:0	878.74 > 716.77	140	44	ESI+
PC 34:1	760.59 > 184.07	30	20	ESI+
PC 36:3	784.59 > 184.07	30	20	ESI+
PC C34:2	758.57 > 184.07	30	20	ESI+
PC C36:5	780.55 > 184.07	30	20	ESI+
PC C36:4	782.57 > 184.07	30	20	ESI+
PC C36:2	786.60 > 184.07	30	20	ESI+
PC C36:1	788.62 > 184.07	30	20	ESI+
PC C38:7	804.55 > 184.07	30	20	ESI+
PC C38:6	806.57 > 184.07	30	20	ESI+
PC C38:5	808.59 > 184.07	30	20	ESI+
PC C38:4	810.60 > 184.07	30	20	ESI+
PC C40:8	830.57 > 184.07	30	20	ESI+
PC C40:7	832.59 > 184.07	30	20	ESI+
PC C40:6	834.60 > 184.07	30	20	ESI+
PC C40:5	836.62 > 184.07	30	20	ESI+
PC C40:4	838.63 > 184.07	30	20	ESI+
PE C34:2	716.52 > 575.50	30	20	ESI+
PE C34:1	718.54 > 577.52	30	20	ESI+
PE C36:5	738.51 > 597.49	30	20	ESI+
PE C36:4	740.52 > 599.50	30	20	ESI+
PE C36:3	742.54 > 601.52	30	20	ESI+
PE C36:2	744.55 > 603.54	30	20	ESI+
PE C36:1	746.57 > 605.55	30	20	ESI+
PE C38:7	762.51 > 621.49	30	20	ESI+
PE C38:6	764.52 > 623.50	30	20	ESI+
PE C38:5	766.54 > 625.52	30	20	ESI+
PE C38:4	768.55 > 627.54	30	20	ESI+
PE C40:8	788.52 > 647.50	30	20	ESI+
PE C40:7	790.54 > 649.52	30	20	ESI+
PE C40:6	792.55 > 651.54	30	20	ESI+
PE C40:5	794.57 > 653.55	30	20	ESI+
PE C40:4	796.59 > 655.57	30	20	ESI+
PE(P) 18:0/20:4	752.56 > 361.27	76	24	ESI+
PE(P) 16:0/22:4	752.56 > 389.31	76	24	ESI+
PE(P) 18:1/22:6	774.54 > 385.27	76	24	ESI+
PE(P) 18:0/22:6	776.56 > 385.27	76	24	ESI+
PE(P) 18:1/22:5	776.56 > 387.29	76	24	ESI+
PE(P) 18:0/22:5	778.58 > 387.29	76	24	ESI+
PE(P) 18:1/22:4	778.58 > 389.31	76	24	ESI+
PE(P) 18:0/22:4	780.59 > 389.31	76	24	ESI+
PE(P) 18:1/16:0	702.54 > 313.27	76	24	ESI+

PE(P) 16:0/18:1	702.54 > 339.29	76	24	ESI+
PE(P) 16:0/20:4	724.53 > 361.27	76	24	ESI+
PE(P) 18:1/18:1	728.56 > 339.29	76	24	ESI+
PE(P) 18:0/18:1	730.58 > 339.29	76	24	ESI+
PE(P) 16:0/22:6	748.53 > 385.27	76	24	ESI+
PE(P) 18:1/20:4	750.54 > 361.27	76	24	ESI+
PE(P) 16:0/22:5	750.54 > 387.29	76	24	ESI+
PE(P) 16:1/18:1	700.53 > 339.29	76	24	ESI+
PE(P) 16:1/22:6	746.51 > 385.27	76	24	ESI+
PE(P) 16:1/22:4	750.54 > 389.31	76	24	ESI+
SM C34:2	701.58 > 184.07	30	20	ESI+
SM C34:3	699.58 > 184.07	30	20	ESI+
SM C36:3	727.61 > 184.07	30	20	ESI+

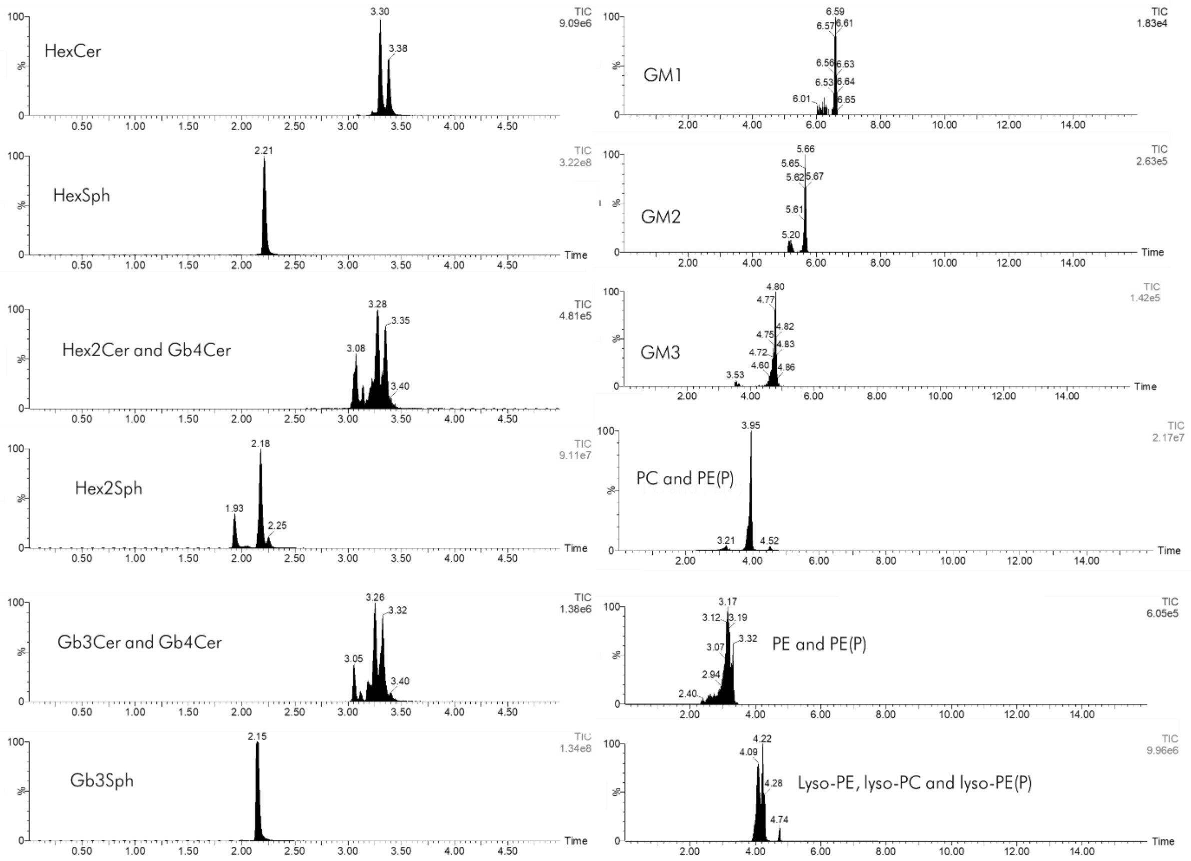

Supplementary Methods Figure S1. Chromatograms from key lipids. *The chromatograms are composed by total ion chromatograms and contain a maximum of 24 species per channel. The x-axis shows the retention time, and the y-axis shows the abundance normalised to percentage of the highest recorded peak.*
