# Supplementary material for: Multi-omic analysis reveals lipid dysregulation associated with mitochondrial dysfunction in parkinson’s disease brain
Source: Nat Commun. 2025 Nov 25;16:10490. doi: 10.1038/s41467-025-65489-2 (PMC12647800; doi:10.1038/s41467-025-65489-2)
Supplement: Supplementary file 2 — Reporting Summary [file 41467_2025_65489_MOESM2_ESM.pdf]

nature portfolio

Corresponding author(s): Wendy Heywood

Last updated by author(s): Sep 19, 2025

Reporting Summary

Nature Portfolio wishes to improve the reproducibility of the work that we publish. This form provides structure for consistency and transparency in reporting. For further information on Nature Portfolio policies, see our Editorial Policies and the Editorial Policy Checklist.

Statistics

For all statistical analyses, confirm that the following items are present in the figure legend, table legend, main text, or Methods section.

n/a	Confirmed	
☐	☒	The exact sample size (*n*) for each experimental group/condition, given as a discrete number and unit of measurement
☐	☒	A statement on whether measurements were taken from distinct samples or whether the same sample was measured repeatedly
☐	☒	The statistical test(s) used AND whether they are one- or two-sided<br>*Only common tests should be described solely by name; describe more complex techniques in the Methods section.*
☒	☐	A description of all covariates tested
☐	☒	A description of any assumptions or corrections, such as tests of normality and adjustment for multiple comparisons
☐	☒	A full description of the statistical parameters including central tendency (e.g. means) or other basic estimates (e.g. regression coefficient) AND variation (e.g. standard deviation) or associated estimates of uncertainty (e.g. confidence intervals)
☐	☒	For null hypothesis testing, the test statistic (e.g. *F*, *t*, *r*) with confidence intervals, effect sizes, degrees of freedom and *P* value noted<br>*Give P values as exact values whenever suitable.*
☒	☐	For Bayesian analysis, information on the choice of priors and Markov chain Monte Carlo settings
☒	☐	For hierarchical and complex designs, identification of the appropriate level for tests and full reporting of outcomes
☒	☐	Estimates of effect sizes (e.g. Cohen's *d*, Pearson's *r*), indicating how they were calculated

Our web collection on statistics for biologists contains articles on many of the points above.

Software and code

Policy information about availability of computer code

Data collection	Human brain tissue samples were obtained from ethically approved biobanks and processed using standardised protocols for lipidomic profiling. Mass spectrometry data were acquired using LC-MS/MS platforms, with consistent instrument settings across batches. Sample meta-data, including clinical diagnosis and post-mortem interval, were recorded and integrated into the analytical pipeline. All data collection procedures adhered to institutional and regulatory guidelines.
Data analysis	Analyses were conducted primarily in Python (version 3.11.11), using SciPy (version 1.9.3), Statsmodels (versions 0.13.5 and 0.14.0), Seaborn (version 0.12.2), Matplotlib (version 3.7.0), UMAP (version 0.5.3), and HDBSCAN. Linear mixed models were implemented via pymer4 (version 0.8.0). Multivariate modelling, including PCA, OPLS, and OPLS-DA, was performed in SIMCA (version 18, Umetrics Sartorius Stedim). Pathway enrichment and Gene Ontology annotations were conducted using DAVID Bioinformatics Resources (2021 build), and network visualisations were created in Cytoscape (version 3.8.0) with yFiles' Organic layout.

For manuscripts utilizing custom algorithms or software that are central to the research but not yet described in published literature, software must be made available to editors and reviewers. We strongly encourage code deposition in a community repository (e.g. GitHub). See the Nature Portfolio guidelines for submitting code & software for further information.

Data

Policy information about availability of data

All manuscripts must include a data availability statement. This statement should provide the following information, where applicable:

- Accession codes, unique identifiers, or web links for publicly available datasets
- A description of any restrictions on data availability
- For clinical datasets or third party data, please ensure that the statement adheres to our policy

The raw targeted chromatograms of the lipid data are available to view and download via the Panorama repository (https://panoramaweb.org/PD_Brain_Lipids.url). Source data are provided with this paper.

Research involving human participants, their data, or biological material

Policy information about studies with human participants or human data. See also policy information about sex, gender (identity/presentation), and sexual orientation and race, ethnicity and racism.

Reporting on sex and gender	Sex-related differences in brain lipid profiles were evaluated using Orthogonal Partial Least Squares–Discriminant Analysis (OPLS-DA) across control samples. No significant differences in lipid abundance were observed between male and female donors (ANOVA cross-validation p = 1), indicating that sex did not contribute substantially to the variance in the dataset.
Reporting on race, ethnicity, or other socially relevant groupings	Race, ethnicity, and other socially relevant groupings were not evaluated in this study. Donor meta-data were limited to age, sex, diagnosis, and brain region, and no stratified analyses by race or ethnicity were performed.
Population characteristics	The study included post-mortem brain tissue samples from individuals diagnosed with Parkinson's disease (PD) and matched controls, spanning eight brain regions. PD cases were stratified by Braak stage into early (Braak 3–4) and late (Braak 5–6) pathology. Sample sizes per region ranged from 5 to 23 for PD and 11 to 16 for controls. Mean ages varied between 70.7 and 79.6 years, with standard deviations typically between 5.9 and 10.6 years. Female representation ranged from 17% to 64% across groups. Age differences between PD and control groups were assessed using Benjamini-Hochberg adjusted Student's two-tailed t-tests, with significance observed in select regions including the cerebellum, frontal cortex, parietal cortex, and temporal cortex.
Recruitment	This study used post-mortem brain tissue samples obtained from established biobanks. No participants were recruited directly by the authors. Sample selection was based on diagnostic classification, Braak staging, and availability of matched control tissue across eight brain regions.
Ethics oversight	post-mortem brain tissue was obtained from the Neurological Tissue Bank, IDIBAPS-HC-Biobanc, Barcelona; Human Brain Tissue Bank, Budapest; UK Parkinson's Disease Society Tissue Bank, Imperial College London; the London Neurodegenerative Diseases Brain Bank, Institute of Psychiatry, King's College, London; Netherlands Brain Bank, Amsterdam; and the Newcastle Brain Tissue Resource. Informed consent was given in all cases. Ethical approval for the study was obtained from the Local Research Ethics Committee of the National Hospital for Neurology and Neurosurgery.

Note that full information on the approval of the study protocol must also be provided in the manuscript.

Field-specific reporting

Please select the one below that is the best fit for your research. If you are not sure, read the appropriate sections before making your selection.

☒ Life sciences ☐ Behavioural & social sciences ☐ Ecological, evolutionary & environmental sciences

For a reference copy of the document with all sections, see nature.com/documents/nr-reporting-summary-flat.pdf

Life sciences study design

All studies must disclose on these points even when the disclosure is negative.

Sample size	No formal sample size calculations were performed. This study utilised post-mortem human brain tissue from existing biobank collections, and sample availability was constrained by diagnostic classification, Braak staging, and regional tissue integrity. All available samples meeting inclusion criteria were analysed to maximise statistical power within these constraints.
Data exclusions	No data were excluded.
Replication	The study employed multiple strategies to ensure reproducibility. Multivariate analyses, including OPLS and OPLS-DA, were validated using cross-validation and permutation testing to assess model robustness. Univariate statistical comparisons were corrected for multiple testing using the Benjamini-Hochberg procedure (FDR = 5% or 10%).
Randomization	Sample preparation and analytical run order were randomised to minimise batch effects. Constrained randomisation was applied during sample list generation to ensure balanced representation of diagnostic groups across batches.

Blinding	Sample preparation was performed under blinded conditions to minimise bias. Diagnostic information was withheld during tissue processing. However, blinding was not maintained during sample list generation and run order assignment, as diagnostic classification was required for constrained randomisation across analytical batches.

Reporting for specific materials, systems and methods

We require information from authors about some types of materials, experimental systems and methods used in many studies. Here, indicate whether each material, system or method listed is relevant to your study. If you are not sure if a list item applies to your research, read the appropriate section before selecting a response.

Materials & experimental systems

n/a	Involved in the study
☒	☐ Antibodies
☒	☐ Eukaryotic cell lines
☒	☐ Palaeontology and archaeology
☒	☐ Animals and other organisms
☒	☐ Clinical data
☒	☐ Dual use research of concern
☒	☐ Plants

Methods

n/a	Involved in the study
☒	☐ ChIP-seq
☒	☐ Flow cytometry
☒	☐ MRI-based neuroimaging

Plants

Seed stocks	N/A
Novel plant genotypes	N/A
Authentication	N/A
